# Cafeteria diet induce changes in blood flow that are more related with heat dissipation than energy accretion

**DOI:** 10.7717/peerj.2302

**Published:** 2016-08-03

**Authors:** David Sabater, Silvia Agnelli, Sofía Arriarán, María del Mar Romero, José Antonio Fernández-López, Marià Alemany, Xavier Remesar

**Affiliations:** 1 Department of Biochemistry an Molecular Biomedicine, Faculty of Biology, Universitat de Barcelona, Barcelona, Spain; 2 CIBER OBN Research Network, Barcelona, Spain; 3 Institute of Biomedicine, Universitat de Barcelona, Barcelona, Spain

**Keywords:** Flux, Cafeteria diet, Rat, Tissue blood flow

## Abstract

**Background.** A “cafeteria” diet is a self-selected high-fat diet, providing an excess of energy, which can induce obesity. Excess of lipids in the diet hampers glucose utilization eliciting insulin resistance, which, further limits amino acid oxidation for energy.

**Methods.** Male Wistar rats were exposed for a month to “cafeteria” diet. Rats were cannulated and fluorescent microspheres were used to determine blood flow.

**Results.** Exposure to the cafeteria diet did not change cardiac output, but there was a marked shift in organ irrigation. Skin blood flow decreased to compensate increases in lungs and heart. Blood flow through adipose tissue tended to increase in relation to controls, but was considerably increased in brown adipose tissue (on a weight basis).

**Discussion.** The results suggest that the cafeteria diet-induced changes were related to heat transfer and disposal.

## Introduction

The cafeteria diets (CD) were devised as a dietary model in which palatability could overcome the intrinsic control of energy intake of experimental animals and induce hyperphagia, consequently, its basis is taste and variety (Sclafani & Springer, 1976). There is a wide variety of formulations of cafeteria diets, all based on these principles, and have been widely used for the study of late-onset hyperphagic obesity and metabolic syndrome in animal models (Sampey et al., 2011), since it induces the increase of fat storage (Rothwell & Stock, 1976). These models have the advantage of being comparable, at least in its basic characteristics (self-selection, excess energy intake), to human obesity induced by energy-dense diets (Romero, Esteve & Alemany, 2006). In general, the lipid intake with CD is grossly increased, while other nutrients are consumed in proportions barely different from controls: most CD are, thus, hyperlipidic (Prats et al., 1989), and their effects are more marked in males (Prats et al., 1989; Coatmellec-Taglioni et al., 2003), not protected by estrogen, and are thus more prone to be affected by glucocorticoids (Bouclaous et al., 2003; Carsia et al., 2008), which in turn compound the obesogenic effect by decreasing the anabolic and protective effects of androgens (MacAdams, White & Chipps, 1986; Retana-Márquez et al., 2003).

In rats, CD increase the accumulation of body fat, but also affect their lean body mass by favoring growth and protein deposition (Harris, 1993; Lladó et al., 1995). The hedonic component of CD initially elicits marked increase in food consumption (Rogers & Blundell, 1984); however, when obesity has been already developed, hyperphagia decreases, often down to normal food intake. Nevertheless, the accumulated excess persists even after normalization of energy intake (Davidson & Garvey, 1993; Coatmellec-Taglioni et al., 2003). The variable presence of sucrose in a number of CD diets can elicit binge eating in humans (Latner, 2008) and in rodents induce similar situations to metabolic syndrome (Santuré et al., 2002), probably because of the additional obesogenic effects of fructose availability (Bocarsly et al., 2010).

All the changes elicited by CD-induced excess energy intake, necessarily influence the interorgan metabolic relationships, changing their ability to control and metabolize substrates (Macedo et al., 2015), the storage of excess energy as reserves (mostly fat), affecting the whole body energy economy to the new pathologic situation. Since dietary-restricted (Nutter et al., 1979) or malnourished rats (Sakanashi, Brigham & Rasmussen, 1987) show decreased heart blood output, in connection with their decreased energy consumption mode, we expected that overfed animals, with excess energy available could show a reverse blood output pattern. The hypothesis was to assume that a higher blood flow in the rat tissues would favor the metabolic interchange and thus help increase energy expenditure and substrate disposal (including storage). Thus, in the present study we have intended to determine whether a common hyperlipidic CD could affect significantly the overall and individual organ blood flow.

## Materials & Methods

### Animal handling conditions

The experimental setup and all the animal handling procedures were carried out in accordance with the guidelines established by the European, Spanish and Catalan Authorities. The specific procedures used in this study were authorized by the Committee on Animal Experimentation and Ethics of the University of Barcelona (Procedure DAAM 6911).

### Animals and diets

Nine week old male Wistar rats (Harlan Laboratories Models, Sant Feliu de Codines, Spain) (initial weight 288 ±2 g) were used. Two groups of 12 animals were randomly selected and were fed *ad libitum* for 30 days with either normal rat chow (#2014 Harlan) or a simplified hyperlipidic cafeteria diet (Esteve et al., 1992b); the groups were named “control” and “cafeteria,” respectively. The rats were housed in 2-rat cages, and were kept in a controlled environment (lights on from 08:00 to 20:00; 21.5–22.5 °C; 50–60% humidity). They had free access to water. The rats in the Cafeteria group were fed with fresh offerings of excess chow pellet, liver pâté spread on cookies, bacon and milk enriched with 300 g/L sucrose and 10 g/L of a protein, vitamin and mineral supplement (Meritene, Nestlé Nutrition, Barcelona, Spain) (Esteve et al., 1992a; Esteve et al., 1992b). Consumption of every item in the CD was recorded daily and converted into nutrient/energy equivalents to establish the actual intake of nutrients in their food selected by the cafeteria group. Body weight was recorded along with food consumption. In the standard diet, 13% of total energy was derived from lipids, 20% from protein and 67% from carbohydrates. In the cafeteria diet, those percentages (mean values) were 43%, 14% and 43%, respectively for the whole period analyzed.

### Permanent cannulations

On day 27, all rats were implanted with two cannulas through the left carotid artery using Intramedic PE-10 polyethylene tubing (Becton Dickinson, Parsipanny, NJ, USA), (0.28 mm id/0.61 mm od) under isoflurane anesthesia. The first carotid cannula was used to draw blood from the descending aorta and the other to inject microspheres directly into the heart outflow as previously described (Ardévol et al., 1998). At 12 h intervals, the viability of the cannulas was checked (without disturbing the animals) by drawing blood up a few mm into the capillary tube, followed by refilling with heparinized saline. On day 30, the rats were injected through the left ventricle cannula with 10^5^ red latex beads (Molecular Probes, Carlsbad, CA, USA) suspended in 0.1 ml of 9 g/L NaCl. At the same time, about 0.2 ml of blood were slowly drawn through the other cannula for exactly one minute. Then, the rats were anesthetized with isoflurane, and larger blood samples were drawn by aortic puncture, euthanizing them. The blood was used to obtain plasma, which was frozen and kept at −80 °C. After sacrifice, the position of the cannulas was checked; no placement errors nor cannula clotting were found. Tissue samples were obtained, frozen, and then maintained at −80 °C. The weights of all organs sampled were also recorded.

### Blood flow analysis

Blood and tissue samples of known volume/weight were digested with 4 M KOH at 25 °C for 24 h with occasional stirring. The samples were filtered through glass-fiber filters (GF/D, 2.5 µm; Whatman, Maidstone, Kent, UK), which retained the colored latex microspheres. The filters were washed with 20 g/L Tween-20 followed by distilled water. Then, the fluorospheres were extracted from the filter with 2.5 ml of etoxyethyl acetate. A fluorimeter (RF1501 Shimadzu, Kyoto, Japan) was used to measure the fluorescence (at 598 nm emission wavelength) under a red excitation light (565 nm), using adequately diluted samples compared against tissue blanks (i.e., pieces of the same tissue of rats from other experiments which had not received fluorescent beads). At least two samples for each tissue and rat were analyzed. The whole procedure was performed in accordance with the instructions provided by the supplied of the fluorescent beads. The number of beads in the tissue samples were estimated from the differential bead extract fluorescence, which was, then, extrapolated to the whole tissue or organ mass. Percent distribution of bead numbers between the different organs was used to determine the distribution of blood flow between the different organs (as percentage), since the total amount of beads injected was known. A sample of the injected material was also analyzed to correct for possible errors in the evaluation of injected bead numbers (Bassingthwaighte et al., 1990).

The calculation of cardiac output was performed by measuring the amount of beads in the blood drawn from the artery for exactly one minute. The estimated bead concentration and the known amount of beads injected allowed the calculation of cardiac output (Closa et al., 1993). In any case, as additional checking, an indirect value of heart output was calculated from body mass (Delp, Evans & Duan, 1998) at rest; the data obtained using both approaches were similar, and thus we used the experimental value for the ensuing calculations.

Absolute blood flows were calculated from the number of beads leaving the heart per unit of time (i.e., absolute heart output), blood volume and the percentage of beads distributed between the different organs of each rat.

### Other analyses

Total body muscle mass was estimated from selective myosin precipitation from the minced and high-concentration lithium chloride-dissolved rat carcass, as previously described (Arola, Herrera & Alemany, 1979). Glucose in plasma with a glucose oxidase kit #11504 (Biosystems, Barcelona, Spain) supplemented with mutarotase (490 nkat/mL of reagent) (Calzyme, San Luis Obispo CA, USA). Mutaroratase was added to speed up epimerization equilibrium of *α*- and *β*-D-glucose and thus facilitate the oxidation of *β*-D-glucose by glucose oxidase (Miwa et al., 1972).

### Statistical analysis

The data were expressed as mean ± standard error. The unpaired Student’s *t* test was used for comparisons between groups (*n* = 12). Statistical analyses were performed using the Prism 5 program (Graph Pad Software, La Jolla, CA USA). Significant differences were stablished at *p* < 0.05.

## Results

After one moth of treatment, control animals weighted 370 ± 15 g and those in the cafeteria-fed group 450 ± 11 g (*p* < 0.05 vs. controls), these values and those of plasma metabolites were similar to other previously described obtained the same dietary model and handling setup (Herrero et al., 1997). Mean daily metabolizable energy intake of control rats was 7.34 ± 0.36 kJ, rats in the cafeteria group ingested 18.4 ± 0.76 kJ/per day (*p* < 0.05 vs. controls). Plama glucose values were similar in both groups (9.97 ± 0.18 mM for controls and 9.68 ± 0.33 mM for cafeteria group).

Table 1 presents the effects 30-day exposure to the cafeteria or standard chow diet on relative organ weight, blood flow and cardiac output. The only significant individual differences in relative organ weight between both groups corresponded to brain, large intestine, lungs and a couple of white adipose tissue sites. The rest of organs showed remarkably similar weights in spite of a slight trend of cafeteria diet-fed rats to increase their size in, in accordance with their higher body size.

**Table 1:** Organ and tissue weight and blood flow of rats fed a cafeteria diet for one month compared with controls on a standard rat chow diet.

Tissue/organ	Control diet			Cafeteria diet			Absolute blood flow ratios: cafeteria/control	*P* values		
	Organ/tissue weight (%bw)	Relative blood flow (mL/min g)	Absolute blood flow (mL/min)	Organ/tissue weight (%bw)	Relative blood flow (ml/min g)	Absolute blood flow (mL/min)		Weight	Relative flow	Absolute flow
Skeletal muscle	39.51 ± 2.41	0.29 ± 0.029	37.7 ± 6.3	39.23 ± 3.11	0.31 ± 0.042	56.0 ± 5.1	1.49	NS	NS	0.049
Skin	15.14 ± 0.63	0.48 ± 0.19	26.3 ± 6.3	13.27 ± 0.64	0.12 ± 0.03	6.8 ± 1.7	0.26	NS	NS	0.014
Liver (arterial)	2.72 ± 0.11	0.24 ± 0.067	2.27 ± 0.64	2.67 ± 0.18	0.56 ± 0.11	5.34 ± 0.60	2.35	NS	0.032	0.006
Liver (total)			14.6 ± 3.98			17.2 ± 3.07	1.18			NS
Small intestine	0.82 ± 0.02	1.77 ± 0.75	8.47 ± 3.57	0.69 ± 0.06	2.34 ± 0.2	8.65 ± 1.90	1.02	NS	NS	NS
Kidneys	0.59 ± 0.02	4.56 ± 1.23	9.43 ± 2.46	0.54 ± 0.02	4.77 ± 0.69	10.8 ± 1.7	1.15	NS	NS	NS
Brain	0.53 ± 0.01	0.65 ± 0.24	1.27 ± 0.47	0.45 ± 0.02	1.09 ± 0.31	2.09 ± 0.61	1.65	0.005	NS	NS
Large intestine	0.41 ± 0.02	1.46 ± 0.37	2.17 ± 0.59	0.31 ± 0.01	2.29 ± 0.38	2.88 ± 0.46	1.33	0.001	NS	NS
Lungs	0.39 ± 0.01	0.47 ± 0.11	0.60 ± 0.10	0.31 ± 0.01	1.08 ± 0.41	1.49 ± 0.24	2.48	<0.001	NS	0.006
Stomach	0.34 ± 0.02	0.91 ± 0.3	1.14 ± 0.38	0.34 ± 0.02	1.1 ± 0.15	1.60 ± 0.26	1.40	NS	NS	NS
Heart	0.26 ± 0.01	0.34 ± 0.18	0.31 ± 0.17	0.26 ± 0.01	0.81 ± 0.38	0.94 ± 0.22	3.03	NS	NS	0.043
Pancreas	0.14 ± 0.003	1.68 ± 0.46	0.62 ± 0.16	0.12 ± 0.003	1.62 ± 0.41	0.80 ± 0.18	1.29	NS	NS	NS
Adrenal glands	0.020 ± 0.005	3.12 ± 0.67	0.22 ± 0.04	0.017 ± 0.004	5.11 ± 0.77	0.35 ± 0.04	1.59	NS	NS	NS
Interscapular BAT	0.11 ± 0.002	0.21 ± 0.074	0.073 ± 0.021	0.11 ± 0.001	0.59 ± 0.22	0.30 ± 0.09	4.11	NS	NS	0.034
Subcutaneous WAT	1.65 ± 0.03	0.12 ± 0.049	0.70 ± 0.30	2.17 ± 0.02	0.084 ± 0.036	0.77 ± 0.33	1.10	<0.001	NS	NS
Mesenteric WAT	0.92 ± 0.02	0.27 ± 0.038	0.49 ± 0.16	0.92 ± 0.02	0.18 ± 0.06	0.71 ± 0.25	1.46	NS	NS	NS
Retroperitoneal WAT	0.72 ± 0.02	0.062 ± 0.02	0.18 ± 0.05	1.11 ± 0.01	0.039 ± 0.008	0.35 ± 0.18	1.94	<0.001	NS	NS
Epididymal WAT	0.42 ± 0.01	0.093 ± 0.04	0.12 ± 0.06	1.06 ± 0.02	0.041 ± 0.008	0.30 ± 0.13	2.50	<0.001	NS	NS
Pericardial WAT	0.07 ± 0.007	0.40 ± 0.125	0.088 ± 0.029	0.07 ± 0.004	1.79 ± 0.51	0.45 ± 0.11	1.16	NS	0.024	0.009
Sum of 5 WAT sites	3.84 ± 0.02		1.34 ± 0.25	5.31 ± 0.04		2.29 ± 0.34	1.71	<0.001		0.045
**Cardiac output**			**111 ± 10**			**116 ± 14**				**NS**

**Notes.**

Values are presented as means ± sem.

Liver (total) = Σ liver (arterial) + small intestine + pancreas + stomach + large intestine; BAT = brown adipose tissue; WAT = white adipose tissue.

Statistical significance of the differences between groups was estimated with Student’s *t* test.

The cardiac output data were similar in both dietary groups. However, in absolute terms, blood flows were higher in most organs and tissues of cafeteria group compared to those in the control group but their weight/size were also higher. The ratios of blood flow for each organ (irrespective of size) between the cafeteria and control groups were higher than 1 for all tissues, except for skin, with IBAT (x4), heart (x3) and lungs (x2.5) showing the maximal differences between both dietary groups. The maximum differences observed were in the interscapular brown adipose tissue mass, which irrigation was more than four-fold higher in the cafeteria than in controls. This contrasts with the case of skin, which, in cafeteria group, only received about one fourth of the blood than controls. In white adipose tissue, when compared on a tissue weight basis, the only significant increase in irrigation corresponded to the small but highly active pericardial WAT mass, followed (in decreasing order) by epididymal, retroperitoneal and mesenteric sites, which did not show significant increases in cafeteria vs. control groups in spite of their larger size. However, the combined total adipose mass showed a significant increase in blood flow.

In the liver, experimental microsphere data represent only the arterial inflow, which was higher in cafeteria than in the control group. Total liver blood flow, which can be calculated adding the arterial inflow to the plus portal flow data (i.e., approximately the sum of pancreas, stomach, intestines and mesenteric WAT) did not show, either, differences between both groups.

When the absolute blood flows were corrected by weight, most of the differences described above disappeared, leaving only those for pericardial adipose tissue and liver arterial, but not total, blood inflow. These data suggest that the cafeteria diet altered only partialy, but significantly, blood flow distribution; the differences observed being largely a consequence of different tissue or organ relative mass, ultimately a consequence of excess energy availability and deposition.

## Discussion

The cafeteria diets are essentially hyper-energetic, tipically hyperlipidic, with mean protein and total carbohydrate intakes proportionally not different from controls fed standard rat chow (Prats et al., 1989; Ferrer-Lorente et al., 2007). As expected, a one-month exposure to the cafeteria diet caused overfeeding and resulted in higher body weights, which translated into a marked increase in adipose tissue depot size and higher muscle mass than those of controls. This is in agreement with previous studies showing that cafeteria (and other high-energy, self-selected tasty hyperlipidic diets) increased fat deposition (Sclafani & Springer, 1976), growth, protein accrual (Harris, 1993; Esteve et al., 1992a) and, also, increased energy output (Ma & Foster, 1989). Glucose plasma levels did not show differences caused by dietary treatment, as a direct consequence of increase in plasma glucose caused by anaesthesia (Zuurbier et al., 2008).

The comparison of the patterns of distribution of blood flow between the control and cafeteria groups agrees with the increase in the importance of brown adipose tissue-related thermogenesis (Tulp, Frink & Danforth, 1982; Ma & Foster, 1989) since this tissue blood flow in was almost four-fold higer in cafeteria group then in controls. Thermogenesis was essentially based on the uncoupling of the mitochondrial proton inflow (used to drive the synthesis of ATP via the directional ATPase system) generated in the oxidative part of the respiratory chain because of the adrenergic activation of a specific uncoupling protein in the inner mitochodrial membrane (Ricquier et al., 1984; Rial & González-Barroso, 2001). This mechanism allows dissipating energy as a heat, and its presence is magnified in small mammals such as rodents, with a high surface to volume ratio.

The blood flow of heart and lungs were also higher in the cafeteria group, which contrasts with the similar heart output of both groups. Consequently, more oxygen was available to maintain the activity of the heart in the rats of cafeteria group, despite showing a similar pumping effectiveness than in controls. The same can be said of lungs. However, the skin blood flow in cafeteria rats was greatly reduced compared with controls. We know that cafeteria diets increase the body energy expenditure and heat output (Ma & Foster, 1989), and, in rats, most of this heat is eliminated through evaporation, largely throughout the respiratory tract rather than from the loss through the skin surface observed in larger mammals as man, by means of higher blood circulation (conduction, radiation, convection). This lowered skin blood flow has been previously observed in other metabolic conditions in which energy expenditure was increased, such as active exercise in obese rats, in which skin circulation was maintained in lean but decreased in obese rats (Ardévol et al., 1998). This suggests that heat loss through skin radiation in rats may be inversely related to body size (including changes in fat stores), thus favoring lung evaporation as main heat-loss control mechanism. Probably, this form of control is more flexible and has less thermic inertia and effectiveness than the loss of heat though a skin evolutively prepared (at least in small, furry mammals) to prevent heat loss. The data on blood flow presented here, under standard, i.e., non-exercise, conditions, supports the postulated heat loss control mechanism shift.

The similarity of blood flows to the liver suggests that the intestine is already efficient organ for the extraction of nutrients, nutrients does not need additional blood (essentially oxygen input and substrates output) supply compared with controls. This may be, in part, a consequence of the global easier digestibility overall of the non-lipid components of the fiber-laden chow compared with the heavy disaccharide content of the CD. The CD energy density is larger than that of the control diet, but the volume of solids actually ingested was not much different between both groups.

The trend to increase blood flow in white adipose tissue suggests a harmonic growth pattern affecting all of them, which is probably a consequence of being part of a single disperse organ (Cinti, 2005), but also by the uniform metabolic response to excess energy. This includes fairly uniform distribution of fat stores in adipose and non-adipose tissues (Romero et al., 2014).

The higher blood flow to striated muscle observed in the cafeteria group is in agreement with a steadier (and increased) supply of substrates to sustain muscle growth and maintenance, coincident with the described increase of amino acid availability (Herrero et al., 1997) and body protein accrual of cafeteria diet-fed rats.

The ingestion of a self-selected hyperlipidic cafeteria diet induces the modification of energy partition (Sclafani & Springer, 1976), insulin resistance (Davidson & Garvey, 1993), accumulation of fat (Rogers & Blundell, 1984; Harris, 1993), increased energy expenditure (Tulp, Frink & Danforth, 1982; Ma & Foster, 1989) and decreased production of urea (Barber et al., 1985). To this list we should add that after a relatively short-time (one month) of exposure to the diet, thermogenesis is most likely kept high, but blood flow distribution is markedly changed in a way most likely related (at least in part) to the disposal of excess heat (i.e., lungs’ evaporation vs. skin radiation and conduction) and to support normal function and body groth and accrual of protein and fat.

## Abbreviations

BAT: Brown Adipose Tissue
WAT: White Adipose Tissue

## Additional Information and Declarations

### Data Availability

The following information was supplied regarding data availability: Dipòsit Digital de la Universitat de Barcelona:
http://hdl.handle.net/2445/96861.
